# Fully integrated photoacoustic microscopy and photoplethysmography of human *in vivo*

**DOI:** 10.1016/j.pacs.2022.100374

**Published:** 2022-05-20

**Authors:** Joongho Ahn, Jin Woo Baik, Yeonggeon Kim, Karam Choi, Jeongwoo Park, Hyojin Kim, Jin Young Kim, Hyung Ham Kim, Sung Hyun Nam, Chulhong Kim

**Affiliations:** a Departments of Electrical Engineering, Convergence IT Engineering, and Mechanical Engineering, School of Interdisciplinary Bioscience and Bioengineering, and Medical Device Innovation Center, Pohang University of Science and Technology, Pohang-si, Gyeongsangbuk-do 37673, Republic of Korea; b Samsung Advanced Institute of Technology, Samsung Electronics Co. Ltd., Suwon-si, Gyeonggi-do 16678, Republic of Korea

**Keywords:** Blood vessel, Vascular movement, Blood volume change, Pulsation, Heartbeat

## Abstract

Photoacoustic microscopy (PAM) is used to visualize blood vessels and to monitor their time-dependent changes. Photoplethysmography (PPG) measures hemodynamic time-series changes such as heart rate. However, PPG’s limited visual access to the dynamic changes of blood vessels has prohibited further understanding of hemodynamics. Here, we propose a novel, fully integrated PAM and photoplethysmography (PAM-PPG) system to understand hemodynamic features in detail. Using the PAM-PPG system, we simultaneously acquire vascular images (by PAM) and changes in the blood volume (by PPG) from human fingers. Next, we determine the heart rate from changes in the PA signals, which match well with the PPG signals. These changes can be measured if the blood flow is not blocked. From the results, we believe that PAM-PPG could be a useful clinical tool in various clinical fields such as cardiology and endocrinology.

## 1 Introduction

Photoacoustic imaging (PAI) excels in visualizing biomolecules by exploiting their high optical absorption without requiring any exogenous contrast agent [1], [2], [3], [4], [5], [6], [7]. Thus, the capability of PAI renders it useful for imaging blood vessels and monitoring hemodynamics by highlighting hemoglobin at visible wavelengths [8]. In addition, PAI enables multiscale imaging from microscopy to clinical applications, depending on which optical and ultrasonic subsystems are combined [9], [10], [11], [12], [13], [14], [15]. Most clinical studies have been conducted in the form of photoacoustic tomography with a high intensity pulsed laser and medical ultrasound machines that are widely used in hospitals and clinics [16], [17], [18], [19]. As another form of PAI, photoacoustic microscopy (PAM) is effective at high-resolution imaging by tightly focusing light [20]. Despite the high-resolution imaging capability of PAM, its relatively slow imaging speed has been pointed out to be a limiting factor for its widespread applications in clinical settings. With recent advances in high-speed scanning, PAM systems with B-scan rates as high as a few hundred hertz have been actively explored [21], [22], [23], [24]. PAM’s ability to perform high-speed and high-resolution imaging (within a few seconds per cubic millimeters and a resolution of a few micrometers) has enabled hemodynamic monitoring studies such as those on external stimulation, drug responses, vascular diseases, and regenerative medicine [25], [26], [27], [28].

On the other hand, photoplethysmography (PPG) has been widely used for measuring hemodynamic features in the time domain, such as heart rate (HR), HR variability, and blood pressure, owing to its high signal-to-noise ratio (SNR) with high sampling rates. Subtle changes in pulse wave morphology in the time domain can be measured and analyzed to reveal hidden hemodynamic features using PPG. The variation in blood volume has generally been accepted as the origin of PPG signals by consensus, rather than direct experimental evidence [29]. Limited visual access to dynamic changes in blood vessels has prohibited a comprehensive understanding of the origin of PPG signals. Although the volumetric model can explain a majority of experimental PPG observations, other complementary mechanisms that can occur simultaneously have not been clarified.

In this study, we propose a fully integrated PAM-PPG system that acquires PA images and PPG signals in parallel. The proposed system has the potential to provide new opto-physiological evidence and further insights on various hemodynamic phenomena including a more thorough understanding of PPG signals. This is achieved by combining PAM’s high-quality imaging capability and PPG’s high SNR signals with a high temporal resolution over a sampling volume.

## 2 Materials and methods

### 2.1 Fully integrated photoacoustic microscopy and photoplethysmography

**Fig. 1:**
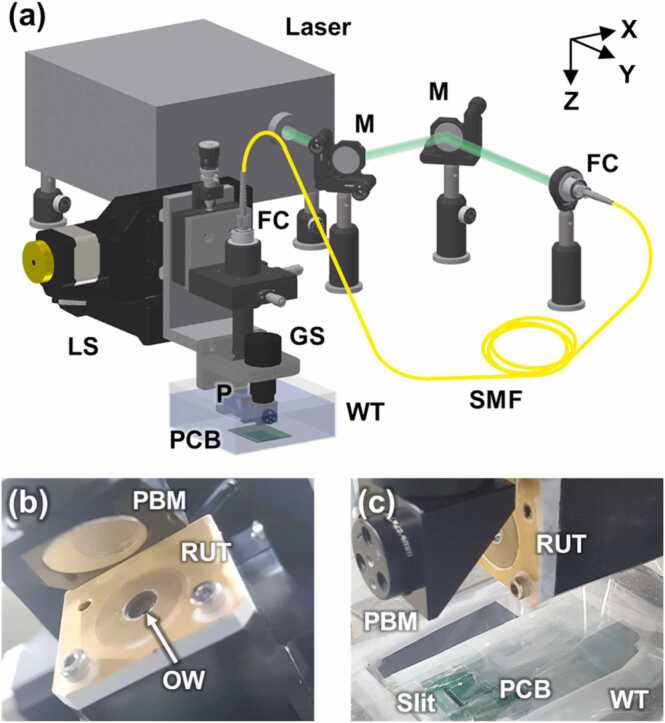
(a) Graphical representation of the combined PAM-PPG system. (b–c) Photographs of (b) the flat ring-shaped ultrasound (US) transducer, parabolic mirror, and PPG PCB wrapped in plastic wrap for waterproofing and attached on water tank. M, mirror; FC, fiber coupler/collimator; SMF, single-mode optical fiber; P, prism; GS, galvanometer scanner; WT, water tank; LS, linear stage; RUT, ring-shaped US transducer; OW, optical window; PBM, parabolic mirror; and PCB, printed circuit board.

The PAM-PPG system is shown schematically in Fig. 1. This PAM is an optical-resolution (OR) mode for high resolution to resolve blood vessels, and such PAM is commonly referred to as OR-PAM. Light for PAM is delivered from a 532 nm pulsed laser (AWAVE 532-1W-10K, Advanced Optowave, Ronkonkoma, NY, USA) to a scanning head through a single-mode fiber (P1-460B-FC, Thorlabs, Newton, NJ, USA) and is then collimated by a fiber coupler/collimator (TC12FC-543, Thorlabs, Newton, NJ, USA). The collimated beam is reflected by a right-angle prism mirror (MRA10-P01, Thorlabs, Newton, NJ, USA) and then passes through an optical window with a diameter of 5 mm (43–365, Edmund Optics, Barrington, NJ, USA) attached to the exit side of the central hole of a customized 15 MHz flat ring-shaped US transducer (5.1 mm inner diameter and 12 mm outer diameter). The laser beam is reflected by an off-axis parabolic mirror (MPD019-P01, Newton, Thorlabs, NJ, USA) to focus on the imaging area. The US transducer and the parabolic mirror are submerged in a water tank. The optical beam irradiates the sample beneath the water tank, where it is closely pressed against a thin plastic membrane. The PA waves generated from the sample return to the US transducer through the same path used by the optical transmission and are detected by the US transducer. The PA signals are amplified by a 50 dB amplifier (PE15A1013, Pasternack, Irvine, CA, USA) and converted to digital signals by a waveform digitizer card (ATS9350, Alazar technologies, Pointe-Claire, QC, Canada) with a 500 MS/s sampling rate. To achieve high-speed imaging, the parabolic mirror is fixed on a galvanometer scanner (GVS011, Thorlabs, Newton, NJ, USA). The above parts for PA imaging are installed on two linear motorized stage (PLS-85, Physik Instrumente, Germany) to structurally align with the PPG components described below.

**Fig. 2:**
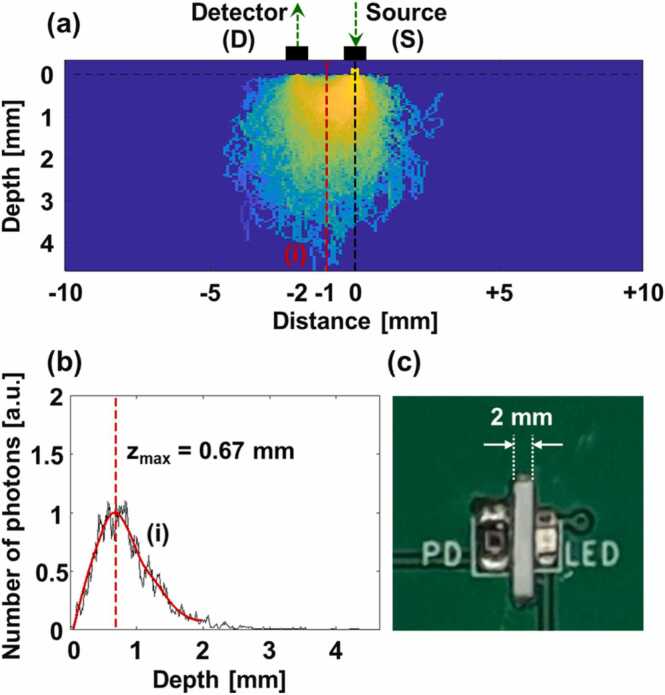
(a) Monte Carlo simulation of photon transfer from a light emitting diode (LED) light source to a photodiode (PD) detector at a distance of 2 mm. (b) Depth profile of red dotted line (i) in (a). (c) 2 mm slit between the LED and the PD in a printed circuit board for photoplethysmography.

A PPG printed circuit board (PCB) composed of a light emitting diode (LED, VLMTG1400, Vishay Semiconductors, Malvern, PA, USA) with 532 nm and a silicon-based photodiode (PD, SFH2716, Osram Opto Semiconductors, Regensburg, Germany). Herein, the green light was selected by considering two prior knowledge: (1) the light sources with visible wavelengths show more dynamic changes between systole and diastole than those with near-infrared wavelengths [30]; (2) the sensitivity of the PD is over 80% on 500–700 nm range. The PCB was located under the water tank membrane to obtain PA and PPG signals simultaneously. The pulsed light and PA waves pass through a slit in the PCB between the LED and the PD. To select an appropriate slit distance, a Monte Carlo simulation was performed on a seven-layered skin model [30]. Fig. 2a represents the traces of photons starting at the source and reaching to the detector. When the distance between the source and the detector is 2 mm, a large amount of LED light can be delivered to approximately 0.67 mm from the surface, at the PAM focus (Fig. 2a–b). Thus, the PAM imaging and PPG sensing depths are co-axially aligned. Fig. 2c is a photograph of the part of the PPG PCB, which shows the 2 mm slit distance between the LED and the PD. The PD signals are amplified by cascaded amplifiers (OPA2380, Texas Instruments, Dallas, TX, USA & ADA4522, Analog devices, Wilmington, MA, USA) and are acquired by an I/O device (PCIe-6321, National Instruments, Austin, TX, USA).

**Fig. 3:**
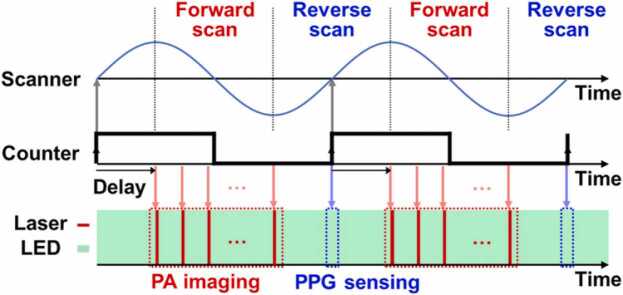
Timing diagram for simultaneous photoacoustic (PA) imaging and photoplethysmographic (PPG) sensing. PA imaging and PPG sensing are performed during forward and reverse scans, respectively, which are fully synchronized by a counter.

To match the sampling intervals of PAM and PPG, a fully synchronized sequence is programmed in the I/O device (Fig. 3). First, a counter with the frequency of the PAM imaging speed is created to use as a reference for triggering. Subsequently, three operations are synchronized on the positive edge of the reference counter: (1) the sinusoidal waveform for a galvanometer scanner with no delays, (2) the triggers for the PD signal acquisition with no delays, and (3) the triggers for the pulsed light irradiation and the PA signal acquisition, with a delay of one-fourth the period of the reference counter. From the synchronized sequence, the PA and PD signals are acquired during forward and reverse scanning, respectively. The acquired PA and PD signals are transferred to our customized program made using LabVIEW (National Instruments, Austin, TX, USA) and are saved on a data storage unit. Signal processing, image visualization, and data analysis are performed using MATLAB (MathWorks, Natick, MA, USA).

Based on the above development, the lateral and axial resolutions for PA imaging are 2.5 µm and 68.4 µm, respectively (Supplementary Fig. 1). Thanks to the short focal length of the parabolic mirror, the lateral resolution is better than 5 µm from the configuration with the objective lens and opto-ultrasound beam combiner in the previous works. On the other hands, the scannable range is about 1 mm, which is narrower compared the previous works due to the trade-off relationship between lateral resolution and scanning range. The maximum scanning speed of PA imaging is limited on 100 Hz, due to the weight of the parabolic mirror although the galvanometer scanner itself can operate over 200 Hz. The PPG signals on the area of fingers and cuticles were up to 5 V, which is large enough to saturate the supplying voltage of the PD. On the in vivo experiments described below, the A and B scan rates of PA imaging were 20 kHz and 50 Hz, respectively, and the sampling frequency of PPG signals was 50 Hz. The 50 PA images and 50 PPG signals per second were acquired by turns during forward and reverse scanning, respectively, which are significantly frequent compared to healthy human’s heart rate of about 1 Hz. Thus, it was considered that the PA and PPG signals were obtained from same targets.

### 2.2 *In vivo* PA imaging and PPG sensing in a human

For human experiments, all experimental procedures were approved by the Institutional Review Board (IRB) of Pohang University of Science and Technology. We recruited three healthy volunteers, explained the procedures thoroughly, and received their informed consents. The subjects and the experimenter wore laser safety glasses and clothes to protect their eyes and body. Before the imaging experiments, we wrapped the PPG-PCB system in a thin plastic film for waterproofing and attached the PCB under the water tank. Next, we filled the gap between the water tank and the PCB with US gel. With the subjects’ finger placed on a customized finger holder, we lifted the holder to bring the finger into close contact with the LED and the PD. The subjects were then instructed to maintain this positioning. After all experimental preparation, PA imaging and PPG sensing was performed. To get reliable data from the unavoidable motion of the subjects, each experiment was performed for about 1 min, which is quite longer than 10 s of data required for analysis. Then, the analysis was conducted using the PA images with few motion artifacts. When the blood flow was blocked temporarily, the upper arm was compressed at 150 mmHg using an aneroid sphygmomanometer (HS-2000, Green Cross Medical Science, Republic of Korea). The measured laser fluence was 12 mJ/cm^2^, below the 20 mJ/cm^2^ maximum permissible exposure for skin safety specified by the American National Standards Institute. As expected, no laser-induced burns were observed on the fingers.

## 3 Results

### 3.1 *In vivo* simultaneous PA imaging and PPG sensing

**Fig. 4:**
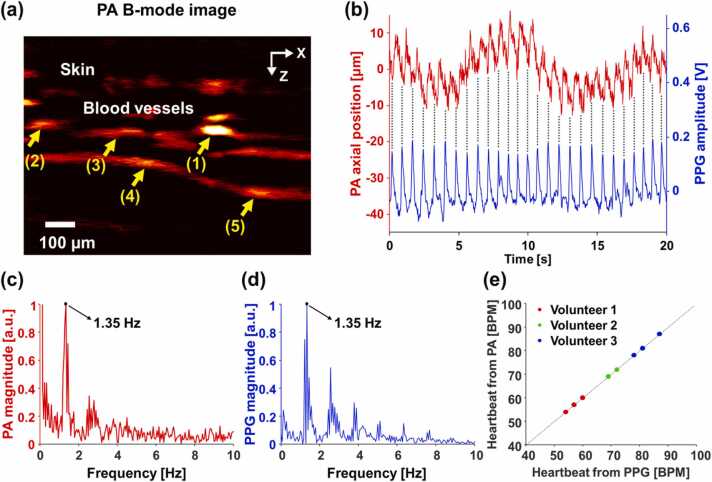
(a) Cross-sectional PA B-mode image of a human finger. The 5 blood vessels indicated by yellow arrows were tracked to quantify their averaged movement. Quantifications of (a) vascular movement by PAM and change in PPG signals over time. (c, d) Frequency responses of (b) and their dominant frequencies of 1.35 Hz. (e) Comparison of the heart rates obtained from vascular movement and the change of blood volume in three healthy volunteers from three repeated experiments. See Supplementary Video 1.

To demonstrate the feasibility of the PAM-PPG system, we acquired PA images and PPG signals from the fingers of the three volunteers. The cross-sectional PA B-mode image in Fig. 4a clearly shows the skin and the blood vessels, and the vessel movements can be observed in the consecutive PA images (Supplementary Video 1). To quantify the vascular movements from the consecutive PA images, we tracked a single vessel [10]. For the first image, we selected a region of interest (ROI) that included a single blood vessel in one image and found a pixel with the maximum PA signal in the ROI. Subsequently, we repeatedly applied the aforementioned algorithm to the consecutive PA images to determine the pixel’s axial position. By applying the above processing to the 5 blood vessels indicated by the yellow arrows in Fig. 4a, it was confirmed that the blood vessels moved with the same pattern (Supplementary Fig. 2a), and finally the averaged vascular movement of the blood vessels was obtained (red line in Fig. 4b). A comparison of the vascular movement with the PPG signals (blue line in Fig. 4b) reveals that both are periodic and completely in phase, which can be verified by through black dotted lines in Fig. 4b. Further, we analyzed the vascular movements and the PPG signals in the frequency domain and obtained their dominant frequencies of 1.35 Hz, which corresponds to 81 beats per minute (BPM) in HR (Fig. 4c–d and Supplementary Fig. 2b). The two dominant frequencies indicate that the HRs coincide seamlessly, demonstrating that the HR obtained from the vascular movement by PAM agrees well with that obtained from the blood volume by PPG. Moreover, the HRs from the vascular movements in the three healthy volunteers agree well with those derived from the blood volume changes (Fig. 4e). Although the HR of each volunteer differs from experiment to experiment, the HRs measured by PAM and PPG for a specific trial are always identical.

### 3.2 *In vivo* simultaneous PA imaging and PPG sensing with arterial occlusion

**Fig. 5:**
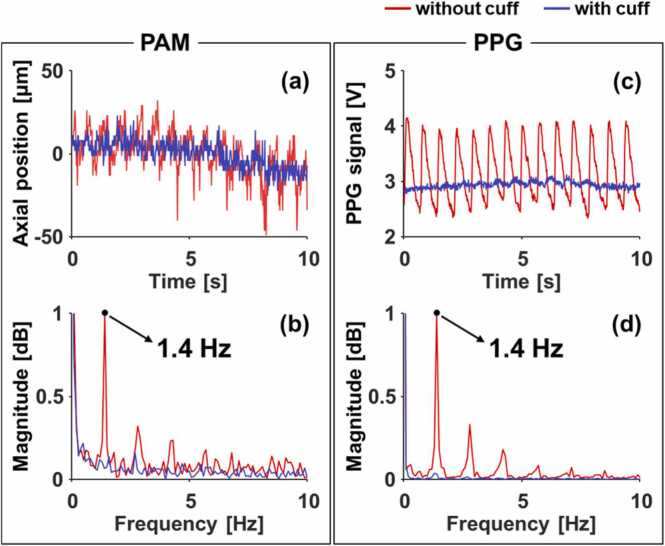
(a) Vascular movements from photoacoustic microscopy (PAM) and (b) photoplethysmographic (PPG) signals under normal and brachial cuffed conditions. (c–d) Frequency responses of (a) and (b).

To investigate the relationship between the vascular movement and the PPG signals further, we conducted experiments of PA imaging and PPG sensing under two different conditions. For a normal condition, the same experiment as described in Section 3.1 was performed. For an abnormal condition with a temporarily blocked blood flow, the upper arm was compressed at 150 mmHg using an aneroid sphygmomanometer. Under the normal condition, the blood vessel moved up and down about 50 µm. In contrast, it did not move under the arterial-occluded condition (Fig. 5a). Even in frequency representations, a dominant frequency of 1.4 Hz could be extracted under the normal condition, but no notable frequency components were present under the arterial-occluded condition (Fig. 5b). Similarly, there was a clear difference between the PPG signals under the two conditions. A distinct periodic pattern could be found in the PPG signals under the normal condition, but it was too weak to find the pattern under the arterial-occluded condition. (Fig. 5c). The difference in the magnitude of the frequency component of 1.4 Hz corresponding to the HR in the two different conditions is more evident in the frequency domain (Fig. 5d). The dominant frequency of the PPG signals obtained under the normal condition was, as expected, 1.4 Hz, being the same as the frequency of vascular movement.

## 4 Discussion and conclusion

The PPG signal is proportional to the change in the optical reflectance as a result of cardiac activity. Increased lumen size of arteries by systole pushes out surrounding tissues and other blood vessels. Consequently, the blood vessels move, and this movement can be observed by continuous PA imaging. It can be inferred that these vascular movements represent pulsation because they are caused by sequential changes started from heartbeat. In contrast, when the blood flow delivered to the finger’s arteries is blocked with brachial compression, neither PPG signals nor vascular movements are observed. In a previous study, human HRs were measured with PAM using a commercial US machine to validate the PA results [31], but that validation was indirect. In our study, the HR deduced from vascular movement in PAM was directly and simultaneously validated with the PPG signal in the combined PAM-PPG system.

Simultaneous PA imaging and PPG sensing are meaningful in that they observe the cardiac activity using different technical methodologies. The PPG signals can be obtained by measuring the changes in the amount of light returning from the irradiated light. In general, because light for PPG is delivered without optical focus, various opto-physiological responses may occur. The main mechanism for generating PPG signals is known as changes in the blood volume, but its principle has not been clearly investigated because light cannot be targeted at a specific biological tissue. By contrast, PAM tightly focuses light and selectively acquires PA signals on blood vessels owing to high optical absorption in hemoglobin, as compared to other biological tissues. From high-resolution PA imaging, we found vascular movements from consecutive blood vessel images, and these movements may change the light path and, consequently, the number of photons reaching the PD. From these perspectives, the vascular movements measured in this study can be considered as one of the mechanisms to perturbate the PPG signals.

The PAM subsystem suggested the new approach to co-align the light and acoustic waves using the flat ring-shaped US transducer and parabolic mirror, replacing the traditional setup with the objective lens and opto-ultrasound beam combiner. The parabolic mirror was the key component by playing multiple roles: (1) focusing the light to targets; (2) collimating the generated PA waves on the targets and direct the waves to the US transducer; (3) steering both light and acoustic waves with the help of the galvanometer scanner. From these features, it made optical and acoustic focal lengths equal, removing the alignment process along the propagating direction of the light and acoustic waves. In addition, by placing the parabolic mirror at the end, it can use a shorter focal length than the traditional setups, which can achieve higher NA and better resolution.

The proposed PAM-PPG system can be improved by implementing multimodal imaging with the US imaging capability to investigate other physical phenomena [32]. A previous US imaging study using a single US transducer and a high-speed scanner captured the dilation and constriction of arteries and tissue deformation by pulsation [33]. Our PAM-PPG system already includes a US transducer and a scanner; thus, the US imaging functionality can be easily implemented [34], [35], [36]. Simultaneous US/PA imaging allows visual observation of arterial pulsation and movement of non-arterial blood vessels, providing us clear evidence. With the recently developed transparent US transducers, such multimodal sensing and imaging may be easily implemented [37], [38]. In addition, the PAM-PPG system has the potentials to provide blood oxygen saturation in different sites through multispectral analysis, and each modality has been separately proved for this purpose [39], [40], [41], [42], [43], [44]. Likewise, the integrated PAM-PPG system can furnish with both capillary and arterial oxygen saturations by employing multi-wavelength light sources. Concurrent monitoring of oxygen saturation in oxygen-supplying and consuming blood vessels could be used to new research such as perfusion studies including both macrocirculation and microcirculations. One caveat is that measuring the HR by monitoring vascular movement is vulnerable to motion by the subject. These motions are clearly represented as low-frequency components in Fig. 4b–c. The human HR is typically between 60 and 120 BPM (1–2 Hz), whereas the motion artifacts that were observed below 1 Hz in Fig. 4c can be minimized by suppressing frequency components lower than 1 Hz.

In this study, we develop an integrated PAM-PPG system that simultaneously acquires PA images and PPG signals. To validate the system, we continuously obtained vascular images and PPG signals from human fingers in vivo. Next, we extracted the HR from the vascular movement (captured by PAM) and directly compared this HR value with that indicated by blood volume changes (captured by PPG). These results were consistent. Further, it was confirmed that the above-mentioned hemodynamic changes could not be observed during the arterial occlusion with the temporarily blocked blood flow. From these results, we believe that the PAM-PPG system could be useful in various clinical applications.

## Declaration of Competing Interest

J. Ahn, Y. Kim, J. Park, and H. Kim declare no competing interests. J. W. Baik was with the Pohang University of Science and Technology and is now an employee of Samsung Electronics, Co. Ltd. K. Choi and S. H. Nam are employees of Samsung Electronics, Co. Ltd. J. Y. Kim and C. Kim have financial interests in Opticho, which, however, did not support this work. H. H. Kim have financial interests in Mirae, which, however, did not support this work. Pohang University of Science and Technology and Samsung Electronics, Co. Ltd. signed a contract to conduct the studies reported here.
