# Supplementary material for: Fully integrated photoacoustic microscopy and photoplethysmography of human in vivo
Source: Photoacoustics. 2022 May 20;27:100374. doi: 10.1016/j.pacs.2022.100374 (PMC9133750; doi:10.1016/j.pacs.2022.100374)
Supplement: Supplementary file 1 — Supplementary material [file mmc1.docx]

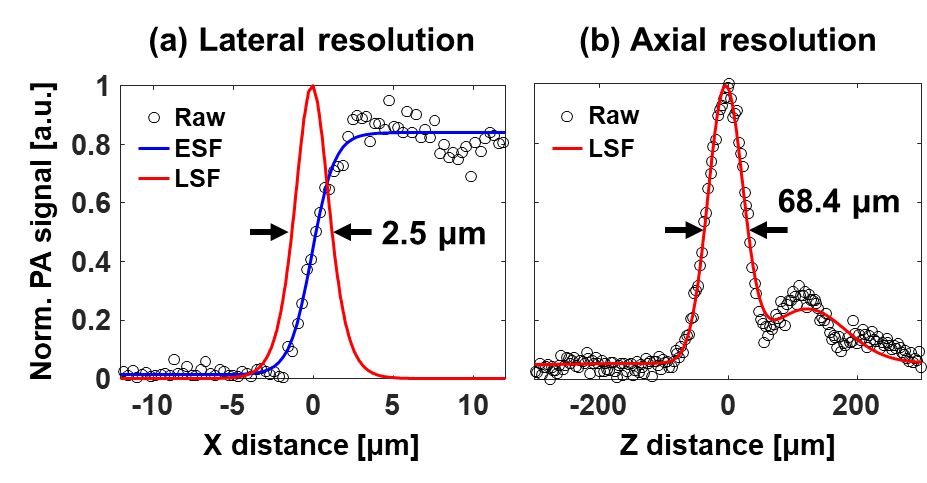


SUPPLEMENTARY FIGURE 1. (a) Lateral and (b) axial resolutions for photoacoustic (PA) imaging. ESF, edge spread function; and LSF, line spread function.


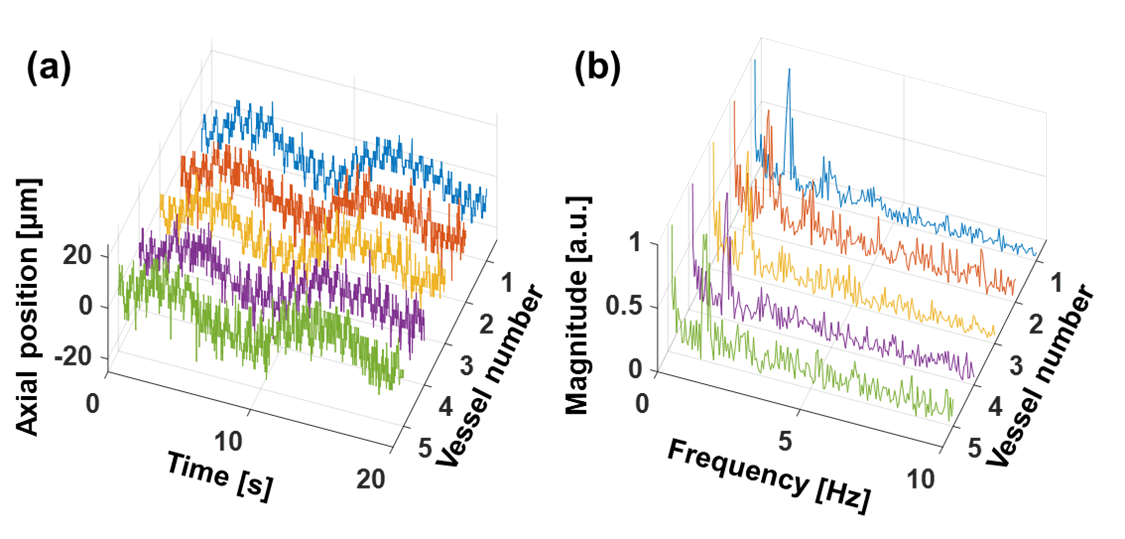


SUPPLEMENTARY FIGURE 2. (a) Vascular movement of 5 blood vessels and (b) frequency responses of (a). The dominant frequencies of the 5 blood vessels are 1.35 Hz.
